# In Vitro Anti-Biofilm Activity of Bacteriophage K (ATCC 19685-B1) and Daptomycin against Staphylococci

**DOI:** 10.3390/microorganisms9091853

**Published:** 2021-08-31

**Authors:** Maria Plota, Eleni Sazakli, Nikolaos Giormezis, Foteini Gkartziou, Fevronia Kolonitsiou, Michalis Leotsinidis, Sophia G. Antimisiaris, Iris Spiliopoulou

**Affiliations:** 1 Department of Microbiology, School of Medicine, University of Patras, 26504 Patras, Greece; plotamaria@yahoo.com (M.P.); kolonits@upatras.gr (F.K.); 2 National Reference Centre for Staphylococci, School of Medicine, University of Patras, 26504 Patras, Greece; giormenik@yahoo.gr; 3 Laboratory of Public Health, School of Medicine, University of Patras, 26504 Patras, Greece; elsazak@upatras.gr (E.S.); micleon@upatras.gr (M.L.); 4 Institute of Chemical Engineering Sciences, FORTH/ICE-HT, Platani, 26504 Patras, Greece; fotini_gartz@yahoo.gr (F.G.); santimis@upatras.gr (S.G.A.); 5 Department of Pharmacy, School of Health Sciences, University of Patras, 26504 Patras, Greece

**Keywords:** staphylococci, biofilm, prevention, phage K, daptomycin

## Abstract

The purpose of the present study was to investigate anti-staphylococcal activity of daptomycin and bacteriophage K, alone or in combination, against biofilm-producers and non-producers *S. aureus* and *S. epidermidis* strains, under biofilm forming and cells’ proliferation conditions. Daptomycin and bacteriophage K (ATCC 19685B1), in different concentrations, were tested against 10 *Staphylococcus aureus* and 10 *S. epidermidis*, characterized by phenotypes and genotypes. The quantitative microtiter plate (crystal violet, CV), methylthiazoltetrazolium (MTT), and growth curve (GC) assays were performed. No statistically significant difference was found between species, whereas daptomycin alone performed better using medium and high concentrations of the drug and bacteriophage K was more active against strains with higher susceptibility, by CV and MTT assays. Best results were achieved using both agents combined in high concentrations. Bacteriophage K was effective within 3.8 and 2.4 h, depending on the concentration used, by the GC assay. Combination of daptomycin with bacteriophage K was more effective against staphylococci, depending on the concentrations used and strains’ susceptibility. Further studies are needed to evaluate if this approach might be a choice for prevention or therapy of biofilm-associated infections.

## 1. Introduction

In recent years major advances have been made in the use of medical devices such as prosthetic joints, artificial implants, contact lenses, peritoneal dialysis, urinary and central venous catheters, endotracheal tubes, mechanical heart valves, and pacemakers. Even though this modern medical technology plays an increasingly important role in the healthcare system, despite all precautionary strategies applied in hospitals to prevent infections, medical devices are vulnerable to contamination and infection [1]. Microorganisms causing device-associated infections belong to the normal microbiota flora of the patient and have the ability to form biofilm [1,2]. The most common aetiologic agents among bacteria are staphylococci and more specifically, *Staphylococcus epidermidis* and *S. aureus* [1,2]. 

Biofilm formation is based on the attachment, proliferation and maturation of bacteria embedded in a matrix of extracellular polymeric substances produced, and a final step of cells’ detachment [2]. Staphylococci are attached to hydrophobic abiotic surfaces by adhesive surface proteins belonging to the Microbial Surface Components Recognizing Adhesive Matrix Molecules (MSCRAMMs), by cell wall anchored proteins and wall teichoic acids. Among the proteins playing an important role in the attachment step are the *S. epidermidis* major autolysin AtlE and the fibrinogen-binding protein (Fbe), whereas, for *S. aureus* the fibronectin binding protein A (FnbA) and the surface protein G, known as adhesin SasG [3,4]. During the second step of biofilm formation, staphylococci upregulate the expression of genes encoding the synthesis of the extracellular polymeric substance forming the matrix consisting of polysaccharides, proteins, teichoic acids, as well as extracellular DNA (eDNA) [2]. Staphylococci encode by the *ica* operon (intercellular adhesion; *icaA*, *icaD*, *icaB*, and *icaC* genes) the production of one main exopolysaccharide called polysaccharide intercellular adhesin (PIA), or poly-*N*-acetylglucosamine (PNAG) [2,4,5]. This extracellular matrix where the bacterial cells are embedded, confers an effective barrier to antibiotics and host defenses. This is accomplished by downregulation of the Agr (accessory gene regulator) system, resulting to a decrease of toxins’ production. Resistance to antimicrobials in biofilm bacterial cells is also due to delayed penetration of the antimicrobial agents through the matrix, to altered growth rate of organisms, and possible other physiological changes [6]. Moreover, biofilm formation acts as an immune evasion mechanism contributing to resistance to neutrophil attacks [1,2]. 

Besides this tolerance to antibiotics due to biofilm aggregates, clinical strains of staphylococci are usually methicillin-resistant carrying a mobile genetic element (Staphylococcal Chromosome Cassette *mec*, SCC*mec*) conferring resistance to beta-lactams (carrying *mecA* or *mecC* genes), but also other resistance determinants. Therefore, these strains are in their majority multi-resistant. Additionally, it has been demonstrated that the rates of genetic exchange are significantly higher in biofilms than planktonic cells [6,7]. Concerning biofilm formation, it has been observed that methicillin-susceptible strains predominantly form *icaADBC*-dependent biofilm, whereas methicillin-resistant strains form biofilms independent of PIA [8]. 

Intervention strategies used to control biofilm formation and device-associated infections include: (a) prevention of contamination, (b) minimization or inhibition of microbial attachment, (c) use of agents that penetrate the biofilm matrix and kill the biofilm-embedded bacteria, or (d) removal of the device [6]. 

In the present study we have focused on the third intervention, testing the activity of daptomycin and bacteriophage K against *S. aureus* and *S. epidermidis* clinical strains. Daptomycin is a cyclic lipopeptide antibiotic that inhibits lipoteichoic acid biosynthesis and oligomerizes cell membrane without complete lysis of bacteria, disrupting multiple aspects of bacterial cell membrane function. Daptomycin is used for the treatment of systemic infections caused by Gram-positive bacteria. Lipopeptides appear to exhibit more potent anti-biofilm action (inhibition or dispersal) than biofilm eradication activity [9]. Daptomycin has been proposed as an alternative therapeutic option in patients with staphylococcal or enterococcal prosthetic joint infections according to Infectious Diseases Society of America (IDSA) guidelines [10,11]. 

Bacteriophages are present in nearly every environment and they play an important role in the control of bacterial population. Numerous reports concern their in vitro application as whole bacteriophages; additionally, bacteriophage-derived endolysins are used as antibacterial agents. However, phages with strictly lytic life cycles are preferred for therapeutic implementation, resulting in killing their host since they have been found to diminish the chances of evolvement phage resistance [12,13].

In this study we have used bacteriophage K, a well-known antistaphylococcal phage that attaches specifically to the cell wall teichoic acids, showing a broader host range and having the ability to lyse *S. aureus* and *S. epidermidis*. Phage K has already been shown to disrupt biofilms produced by *S. aureus* [13,14]. 

Our aim was to investigate the anti-staphylococcal activity of an antibiotic (daptomycin) and a phage with a broader range of hosts (bacteriophage K), alone or in combination, against biofilm-positive and -negative well characterized clinical *S. aureus* and *S. epidermidis* strains, under biofilm forming and cells’ proliferation conditions.

## 2. Materials and Methods

### 2.1. Bacterial Strains

#### 2.1.1. Phenotypic Characterization

Twenty well characterized clinical strains, ten *S. aureus* and ten *S. epidermidis* recovered from clinical specimens of patients with prosthetic device-associated infections were selected and further analyzed (Table 1). Specifically, among *S. aureus* three were recovered from catheter-related bloodstream infections, (CR-BSI), six from periprosthetic tissue infections (PTI), one from central venous catheter-related infection (CVC). Among *S. epidermidis*, six derived from CR-BSI, three from CVC and one from PTI. Identification of strains at species level was initially performed phenotypically by Gram staining, catalase production, coagulase testing (Slidex Staph Plus; bioMérieux, Marcy l’ Etoile, France) and by the Vitek 2 System (GP card, bioMérieux, Marcy l’ Etoile, France). Susceptibility to cefoxitin (FOX), penicillin (PEN), erythromycin (ER), clindamycin (CLI), tobramycin (TOB), gentamicin (GM), ciprofloxacin (CIP), fusidic acid (FA), and sulfamethoxazole/ trimethoprim (SXT) was tested by the disk diffusion method according to EUCAST guidelines [15]. Minimum inhibitory concentration (MIC) of daptomycin (DAP) (Daptomycin ready-made solution 1 mg/mL in DMSO, SBR00014, Sigma-Aldrich, Chemie GmbH, Taufkirchen, Germany) were determined by the broth microdilution method in the presence of Ca^2+^ (50 mg/L in the medium), according to EUCAST guidelines [15]. Methicillin resistance was phenotypically based on the performance of cefoxitin disk diffusion test [15]. Strains showing resistance to at least three classes of antimicrobials were characterized as multi-drug resistant (MDR). All strains were kept frozen in −70 °C in tryptic Soy broth (TSB, Oxoid CM0129, Oxoid Ltd., Wade Road, Basingstoke, Hants RG24 8PW, UK) with 15% glycerol.

The Ethics Committee of the University General Hospital of Patras (UGHP) approved this study and waived the need for informed consent (Approval Number 615).

#### 2.1.2. Genotypic Characterization 

DNA extraction from studied strains was performed using the QIAamp DNA Mini kit (Qiagen, Düsseldorf, Germany, GmbH). Molecular identification to species level was carried out by sequencing analysis of the amplified *tuf* gene [16]. Methicillin resistance was verified by PCR with specific primers for *mecA* gene [17,18]. 

Amplification of *ica* operon genes (*icaA, icaD, icaB*, and *icaC*), and the adhesins’ encoding genes *fnbA* and *sasG* in *S. aureus*, as well as, *fbe* and *atlE* in *S. epidermidis*, was performed by PCRs as described [19,20,21,22,23]. PCR products were analyzed by electrophoresis into 1% agarose gels.

### 2.2. Susceptibility of Strains to Staphylococcus aureus subsp. aureus Bacteriophage K (ATCC^®^ 19685B1^TM^) 

#### 2.2.1. Phage Propagation and Enumeration 

Propagation of bacteriophage K was performed using its host reference strain *Staphylococcus aureus* subsp. *aureus* Rosenbach (ATCC^®^ 19685^TM^) onto 9 cm Brain Heart Infusion agar plates (BHIA, Oxoid CM1136, Oxoid Ltd., Wade Road, Basingstoke, Hants RG24 8PW, UK) by the soft agar overlay method according to standard protocols [24,25]. Enumeration of each propagation’s batch of phage K particles was performed in duplicate, by the small drop assay method with its host ATCC 19685 strain using a total of eight serial 10-fold dilutions of the phage’s stock in Brain Heart Infusion broth (BHIB, LAB049, Lab M Limited 1 Quest Park, Moss Hall Road, Heywood, Lancashire BL9 7JJ, UK) and BHIA as underlay agar in the Petri dishes [26]. 

#### 2.2.2. Strains’ Susceptibility Testing to Bacteriophage K 

It was performed for each strain in duplicate, using a phage suspension of 10^4^ pfu/mL. All staphylococcal strains were inoculated onto BHIA plates and after an overnight incubation, bacterial suspensions equivalent to 0.5 Mac Farland turbidity standard (1.5 × 10^8^ cfu/mL) were prepared in BHIB. From each strain’s suspension, 20 µL were added into 180 µL BHIB together with 20 µL of aforementioned phage suspension; after gentle agitation, 20 µL of the mix were inoculated onto BHIA plates, left to dry, and incubated overnight at 37 °C. Mean values of plaque forming units for each strain were calculated (Table 1). As positive control, the propagating strain ATCC19685 was used and as negative one, a sample without bacterium [26]. 

### 2.3. Biofilm Formation Assay

Biofilm formation was tested in triplicate by the quantitative microtiter plate (crystal violet, CV) assay with minor modifications; briefly, strains were inoculated onto tryptic Soy agar (TSA, Oxoid CM0131, Oxoid Ltd., Wade Road, Basingstoke, Hants RG24 8PW, UK); after an overnight incubation at 37 °C, three colonies were subcultured in 5 mL TSB (Oxoid CM0129, Oxoid Ltd., Wade Road, Basingstoke, Hants RG24 8PW, UK) and incubated for 18 h; after vortexing 2 µL of the suspension were inoculated into 200 µL TSB with 1% glucose (Glucose monohydrate, Ph.Eur., Sigma, Sigma-Aldrich, Chemie GmbH, Taufkirchen, Germany), in 96 well U-bottomed sterile polystyrene plates that were incubated aerobically at 37 °C for 24 h ± 30 min. After washing each well with 300 µL sterile phosphate buffered saline (PBS), plates were heat-fixed at 60 °C for 1 h. Staining was performed with 195 µL crystal violet for Gram staining for 15 min, followed by a washing step with tap water and air dried. Optical density (OD) of each well stained with crystal violet was measured at 570 nm using a microtiter-plate reader (FLUOstar Omega microplate reader, BMG LABTECH GmbH, Germany), after homogeneous resolubilization of the dye with 150 mL of 95% ethanol [27]. The reference strains *S. epidermidis* ATCC 35984 (RP62A), a well characterized slime producing/*ica*-positive strain, and ATCC 12228, a slime-negative/*ica*-negative one, were used as positive and negative controls respectively. The cut-off value (ODc) in each plate was defined as three standard deviations (SD) above the mean OD of the negative control: ODc = average OD of negative control + (3XSD of negative control). Interpretation of results was performed after calculation of the average ODs of all strains, according to the published criteria [27]. 

### 2.4. Activity of Bacteriophage K (ATCC 19685-B1) and Daptomycin against Staphylococci

#### 2.4.1. Crystal Violet Microtiter Plate Assay

Activity of bacteriophage K and daptomycin was investigated by the CV for biofilm forming assay in triplicate for each concentration and strain tested [27]. The method, in each run, was performed as described in Section 2.3, testing every strain alone in 200 µL TSB with 1% glucose, and in the presence of two final concentrations of phage suspensions, 10^4^ pfu/mL (phage low concentration, PLC) and 10^6^ pfu/mL (phage high concentration, PHC). Briefly, in the wells that an antimicrobial agent was tested, in 150 µL of TSB with 1% glucose, 2 µL of bacterial suspension and 50 µL of stock phage suspensions (4 × 10^4^ pfu/mL and 4 × 10^6^ pfu/mL) were added. For daptomycin, four final concentrations were tested in the presence of 1.25 mM Ca^2+^: 0.1 mg/L (daptomycin very low concentration, DVLC), 0.5 mg/L (daptomycin low concentration, DLC), 1 mg/L (daptomycin medium concentration, DMC), and 2 mg/L (daptomycin high concentration, DHC) (stock solutions: 0.4, 2, 4, and 8 mg/L). Both agents were also tested combined in low (PLC-DLC) and high concentrations (PHC-DHC). Experiments were performed in the presence of positive and negative controls (ATCC 35984 and ATCC 12228). Results for each isolate are expressed as mean ODs [27,28,29,30,31]. For every strain positive and negative controls were tested, in triplicate; these were the strain in the medium without any antimicrobial, whereas the negative control was only the medium.

#### 2.4.2. Methylthiazoltetrazolium (MTT) Assay

The MTT assay is based on the uptake and the reduction of 3-[4,5-dimethylthiazol-2-yl]-2,5-diphenyl tetrazolium bromide (MTT, Thiazolyl Blue Tetrazolium, M2128, Sigma, Sigma-Aldrich, Chemie GmbH, Taufkirchen, Germany) by oxidoreductase enzymes of viable cells, to formazan crystals that are dissolved by dimethyl sulfoxide (DMSO). All isolates are tested in triplicate using the same media, concentrations of antimicrobials and controls as for CV method. Briefly, 2–3 colonies of bacteria after an overnight culture onto TSA plates, are inoculated in 5 mL TSB and incubated at 37 °C for 18 h. U-bottomed polystyrene 96-well plates are prepared and incubated overnight at 37 °C. The wells are washed twice with 150 µL PBS followed by the addition of 105 µL MTT (stock solution 98%, final concentration 0.5 mg/mL in PBS) and incubation at 37 °C for 1h. One washing step with 150 µL PBS was performed and 120 µL DMSO ware added with mixing by pipetting until the crystals ware solubilized. Interpretation of results was performed after measurement at 570/620 nm and calculation of the mean OD for each strain [32,33]. Strains’ positive and negative controls were tested as for the CV assay.

#### 2.4.3. Growth Curve (GC) Assay

The method was performed based on a previously published protocol, with modifications [34]. Briefly, after 18 h culture of 2–3 colonies in TSB from a TSA agar plate, 96-well flat-bottomed polystyrene plates were prepared with strains and antimicrobials as described in CV method using TSB (without glucose). ODs were measured at 570 nm, on time “0” followed by incubation at 37 °C and measurements after 2, 4, 6, 8, 10, 12, and 24 h. Mean values of ODs were calculated for each strain. Strains’ positive and negative controls were tested as for the CV assay.

Experiments with daptomycin were not further analyzed by this assay, due to the fact that no differences in ODs were observed comparing strains alone *vs* any intervention with the drug.

In order to examine the kinetics of microbial growth, with or without addition of bacteriophage in low or high concentration, a six-parameter probabilistic model of microbial growth and mortality, developed by Horowitz et al., was employed [35]. It is a double model, constructed from an underlying three-parameter logistic model of division probability, pDiv6[t], and a three-parameter logistic model of mortality, pMort6[t] having different asymptotes, pd_Asym_ and pm_Asym_, steepness parameters, kd and km, and characteristic times, tcd and tcm, respectively. The probabilities of division or mortality are defined by Equations (1) and (2) respectively.

pDiv6[t] = pd_Asym_/(1 +Exp[kd × (tcd − t)]) (1)

pMort6[t] = pm_Asym_/(1 +Exp[km × (tcm − t)]) (2)

The number of cells alive at time t is given by the explicit Formula (3)

(3) $$N\left[t\right]={\;\mathrm{No}\times e}^{\mathrm{pdAsym}\;-\;\mathrm{pmAsym}\;-\frac{\mathrm{pdAsym}\;\mathrm{Log}\left(1+e^{\mathrm{kd}\;\mathrm{tcd}}\right)\;}{\mathrm{kd}}+\frac{\mathrm{pdAsym}\;\mathrm{Log}(1+e^{\mathrm{kd}\;\left(-t+\mathrm{tcd}\right)}\;}{\mathrm{kd}}+\frac{\mathrm{pmAsym}\;\mathrm{Log}(1+e^{\mathrm{km}\;\mathrm{tcm})}\;}{\mathrm{km}}-\frac{\mathrm{pmAsym}\;\mathrm{Log}(1+e^{\mathrm{km}\;\left(-t+\mathrm{tcm}\right)}\;}{\mathrm{km}}}$$

The above formula was applied in the experimental data, to fit and plot the models along with the corresponding probability functions, pDiv6[t] and pMort6[t]. In the place of number of cells, the absorbance units at 570 nm were used instead. Data processing was performed in the Mathematica, v. 12.1.1.0 (Wolfram Research, Inc., Champaign, IL, USA, 2020).

### 2.5. Statistical Analysis

#### 2.5.1. Activity of Bacteriophage K (ATCC 19685-B1) and Daptomycin against Staphylococci (CV and MTT Assays)

ODs of strains alone or with antimicrobials were subtracted from blanks and normalized against the OD of each strain in TSB, in order to be comparable. A two-way ANOVA was conducted to evaluate the effectiveness of bacteriophage and daptomycin, across staphylococci species (*S. aureus* vs. *S. epidermidis*), biofilm formation (positive vs. negative), susceptibility to bacteriophage K (medium, 80–100 pfu, vs. high, >100 pfu) and daptomycin MIC (<0.75 vs. ≥0.75–1 mg/L). The Games-Howell post hoc was employed to perform pairwise comparisons. 

#### 2.5.2. Growth Inhibitory Effect of Bacteriophage 

Staphylococci were divided into two groups—i.e., ‘inhibition’ and ‘no inhibition’—depending on whether the presence of bacteriophage caused mortality probability to dominate over division probability, in the growth curves’ models. The associations of groups of inhibited or non-inhibited strains with independent features of the strains, were assessed via a binary logistic regression model. 

For all tests, the statistical significance was set at a = 0.05. Statistical analysis was performed by IBM SPSS v.25 statistical software (IBM Corp., Armonk, NY, USA).

## 3. Results

### 3.1. Phenotypes and Genotypes of Studied Strains

Among the 20 studied strains, 14 (eight *S. aureus* and six *S. epidermidis*) were biofilm producers (Table 1). From the six biofilm-negative strains, five were associated to CR-BSI and one to CVC infection. Nine strains (two *S. aureus* and seven *S. epidermidis*) were methicillin-resistant carrying *mecA* gene, whereas, eight strains (one *S. aureus* and seven *S. epidermidis*) were MDR. Ten strains (four *S. aureus* and six *S. epidermidis*), all biofilm-positive, carried *ica* operon. Adhesins’ genes carriage is presented in Table 1. All 20 strains were susceptible to bacteriophage K, 10 with medium and another 10 with high susceptibility (80–100 pfu and >100 pfu, respectively). Eleven strains showed daptomycin MIC <0.75 mg/L and nine 0.75–1 mg/L.

### 3.2. Activity of Bacteriophage K and Daptomycin

Species and biofilm formation had no effect on the survival of staphylococci, as the two-way ANOVA revealed (*S. aureus* vs. *S. epidermidis*: *p* = 0.360 and *p* = 0.250 in CV and MTT assays respectively and biofilm-positive vs. biofilm-negative: *p* = 0.926 and *p* = 0.853 in CV and MTT assays, respectively). However, any applied intervention affected ODs (*p* < 0.001 in both assays). All interventions differed from the culture of the strain alone (*p* < 0.001), according to the Games-Howell post-hoc test in both assays, while effectiveness of the intervention increased with increasing concentration of either bacteriophage or daptomycin. Low concentration of bacteriophage (PLC) demonstrated similar activity as compared to either DVLC or DLC, and PHC was as active as DMC or DHC. The use of phage and daptomycin in combination resulted in increased activity, in such a way that the combination of low concentrations (PLC-DLC) performed similarly with either phage (PHC) or daptomycin (DHC) alone in high concentrations, whereas, the combination of phage and daptomycin in high concentrations (PHC-DHC) showed the highest activity. In fact, while PLC reduced absorbance by 37.1% and DLC by 48.9%, their combination resulted in a total reduction of 63.4%, in the CV assay. Similarly, PHC and DHC reduced absorbance by 67.5% and 74.3% respectively and their combination by 85.7%. Analogous results were obtained by the MTT assay. Unfortunately, due to missing values related to the concentrations of phage and daptomycin (when applied alone) that are required to produce equivalent results with the combination treatments, we could not estimate if the effects of the combined treatment are additive of synergistic. Figure S1 depicts the activity order of the antimicrobial interventions by (a) the CV assay and (b) the MTT assay. Statistically significant pairwise differences are indicated. 

**Figure 1:**
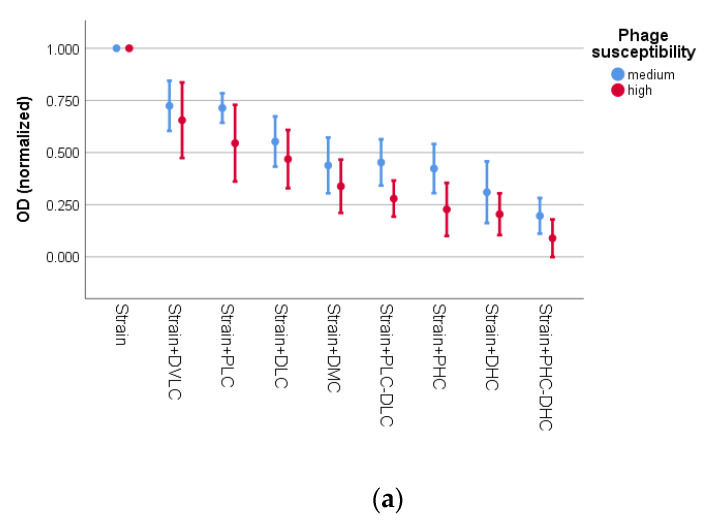

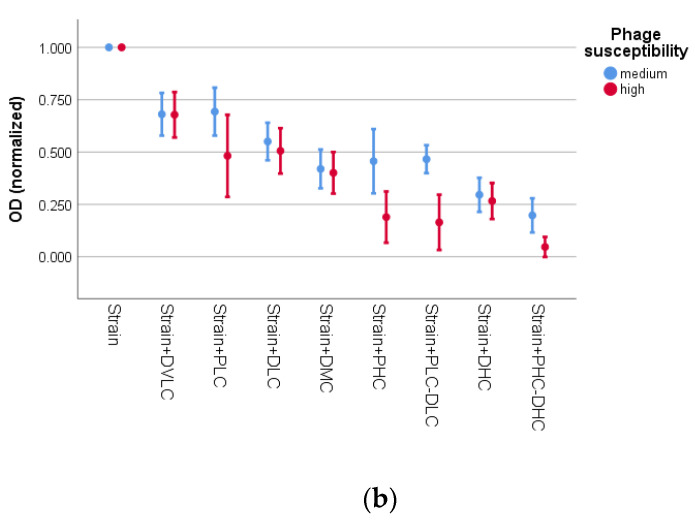
Effect of interventions on normalized OD of strains in relation to susceptibility to phage for (**a**) CV assay and (**b**) MTT assay. Error bars indicate mean OD ± 2 standard error. PLC: 10^4^ pfu/mL (phage low concentration); PHC: 10^6^ pfu/mL (phage high concentration); DVLC: 0.1 mg/L (daptomycin very low concentration); DLC: 0.5 mg/L (daptomycin low concentration); DMC: 1 mg/L (daptomycin medium concentration); DHC: 2 mg/L (daptomycin high concentration); phage susceptibility medium: 80–100 pfu; phage susceptibility high: >100 pfu.

The effect of interventions on staphylococci survival was investigated across susceptibility to phage (medium *vs* high). By the CV assay (Figure 1a), statistically significant differences in ODs by both intervention (*p* < 0.001) and by susceptibility to phage (*p* < 0.001) were observed. For the strains with medium susceptibility to phage (80–100 pfu), activity of PLC was lower than PLC-DLC (*p* = 0.025). Activities of PHC, DHC, and PHC-DHC were comparable. Concerning the strains with high susceptibility to phage (>100 pfu), the Games-Howell post hoc test revealed that PLC and DLC treatments showed a significant difference only with PHC-DHC (*p* = 0.013 and *p* = 0.008, respectively), while PHC yielded similar activity to DMC, DHC and PHC-DHC. In general, the ‘high susceptibility’ group exhibited higher activity as compared to the ‘medium susceptibility’ group, in the interventions involving addition of phage. 

By the MTT assay (Figure 1b), the two-way ANOVA revealed that there was a statistically significant interaction between the effects of intervention and susceptibility to phage on OD (*p* = 0.009), meaning that interventions affect OD in phage-medium susceptibility strains (80–100 pfu) differently than in phage-high susceptibility strains (>100 pfu). To clarify how intervention affects OD in each aforementioned group, we analyzed the two groups separately. In the group of the strains with medium susceptibility to phage, activity of PLC was comparable to that of PLC-DLC, while PHC, DMC, DHC, and PH-DHC performed similarly. In the group of the strains with high susceptibility to phage, PLC, DMC, and DHC showed a significant difference only with PHC-DHC (*p* = 0.026, *p* = 0.001 and *p* = 0.011, respectively). As for the effect of susceptibility to phage across the interventions, it was found again, as in the case of the CV assay, that generally the strains with high susceptibility performed better than those with medium, in the experiments involving addition of phage. Thus, in the treatments with PHC, PLC-DLC, and PHC-DHC, the ‘high susceptibility’ group exhibited higher activity as compared to the ‘medium susceptibility’ group. 

**Figure 2:**
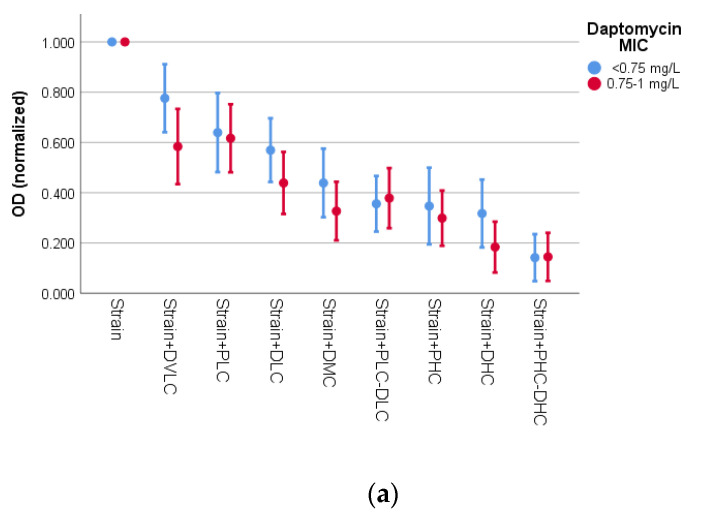

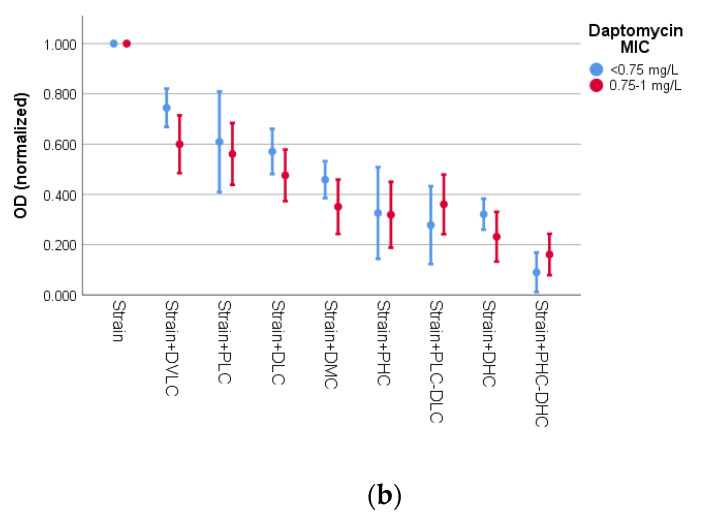
Effect of interventions on normalized OD of strains in relation to daptomycin MIC for (**a**) CV assay and (**b**) MTT assay. Error bars indicate mean OD ± 2 standard error. PLC: 10^4^ pfu/mL (phage low concentration); PHC: 10^6^ pfu/mL (phage high concentration); DVLC: 0.1 mg/L (daptomycin very low concentration); DLC: 0.5 mg/L (daptomycin low concentration); DMC: 1 mg/L (daptomycin medium concentration); DHC: 2 mg/L (daptomycin high concentration).

The last factor we examined was daptomycin MIC. The results of the CV assay (Figure 2a) revealed a statistically significant difference in ODs by intervention (*p* < 0.001) and by daptomycin MIC (*p* = 0.019). To examine the effect of daptomycin MIC, we determined the difference in ODs between interventions at each level of strains’ MIC (<0.75 mg/L vs. 0.75–1 mg/L). Concerning the strains with MIC < 0.75 mg/L, treatment with DVLC did not differ from the culture of the strain alone (*p* = 0.111), but differed from DMC (*p* = 0.046) and the more effective interventions. The PLC, DLC and DMC activities were lower than only PHC-DHC (*p* = 0.001, *p* = 0.001 and *p* = 0.043, respectively). In strains with MIC ranging from 0.75 to 1 mg/L, DVLC exhibited lower activity than DHC (*p* = 0.012) and PHC-DHC (*p* = 0.005), and DLC treatment differed only from PHC-DHC (*p* = 0.036). 

As for the MTT assay (Figure 2b), the factor of daptomycin MIC had no effect on ODs (*p* = 0.164).

### 3.3. Growth Curves 

The fate of microbial growth or mortality, with or without addition of bacteriophage for all studied staphylococci was assessed through the probabilities of division or mortality. The time point, at which the pMort6[t] becomes higher than the pDiv6[t] (and remains such), indicates the time at which the presence of bacteriophage induces massive rapid mortality on staphylococci. In the cases that the pDiv6[t] remains above the pMort6[t] over the course of time, bacteriophage does not provoke any effect or simply causes growth suppression.

**Figure 3:**
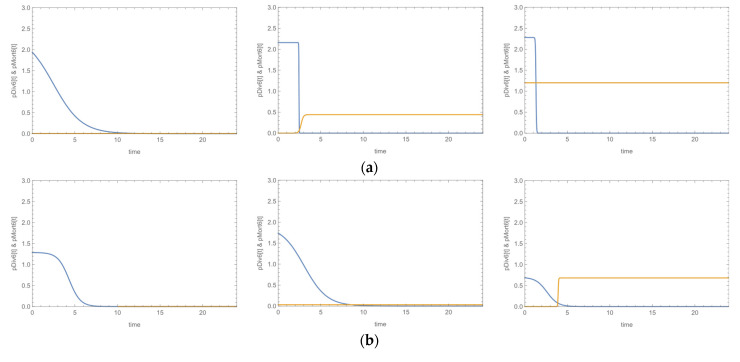

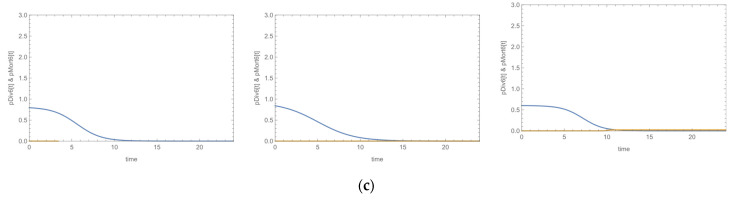
Representative plots of division (blue) and mortality (orange) probabilities of three strains: strain in TSB (left), strain + PLC (10^4^ pfu/mL, center), strain + PHC (10^6^ pfu/mL, right) versus time; (**a**) *S. aureus*, biofilm-positive, both concentrations of bacteriophage produced inhibitory effects; (**b**) *S. aureus*, biofilm-positive, only the high concentration of bacteriophage produced inhibitory effects; (**c**) *S. epidermidis*, biofilm-negative, no inhibitory effect was induced by bacteriophage.

Strains alone exhibited the typical sigmoid growth curves. Addition of bacteriophage to staphylococci produced three possible results: (a) both phage concentrations (PLC, 10^4^ pfu/mL-phage low concentration and PHC, 10^6^ pfu/mL-phage high concentration) induced inhibition of microbial growth; (b) only the high concentration (PHC) affected growth; or (c) none concentration had an effect on microbial growth. Representative plots of division and mortality probabilities versus time for the three cases are presented in Figure 3. The bacteriophage effects are indicated by the intersection of the probability’s plots, if any. The corresponding growth curves are presented in Figure S2. 

Of all 20 strains, nine were affected by the bacteriophage, that caused their mortality irrespective of its concentration, three were affected only by the high concentration of phage and the remaining eight were either suppressed, meaning they reached lower ODs than the corresponding strains alone, or were not at all affected by the bacteriophage. In the strains that bacteriophage had an effect on growth, the domination of mortality was achieved 2–12.5 times earlier than in the culture of the corresponding strain alone. This in practice means that bacteriophage was able to induce inhibition of growth in 3.8 h on average (range 2.5–5.9 h) in the case of PLC, or 2.4 h on average (range 1.0–5.2 h) in the case of PHC, after initiation. These features accounted for the 50. 4% of the ‘variation’ of inhibition, as calculated by the Nagelkerke R^2^ of the model. The results are presented in Table 2.

Susceptibility to phage was significantly associated with inducing inhibitory effects on the growth of staphylococci. The odds of growth inhibition were about 10 times higher in strains with high susceptibility compared to strains with medium susceptibility. Moreover, biofilm-negative strains seem to be more vulnerable to phage than biofilm producers, though this tendency was at the border of statistical significance. 

## 4. Discussion

For the past years, it was common belief that bacteria such as *S. epidermidis* and other coagulase-negative staphylococci (CoNS) were harmless and part of the bacterial flora. Recently, studies in hospitalized patients and patients with indwelling foreign bodies, particularly prosthetic orthopedic implants, intravascular catheters, and cerebrospinal fluid shunts, have shown an emerging problem of health care-associated infections as well as infections caused by these microorganisms on the implanted devices [36,37]. As far as *S. aureus* is concerned, it has always been considered both commensal bacterium and human pathogen. Approximately 30% of the human population is colonized with *S. aureus* [38]. It is considered to be a leading cause of bacteremia and infective endocarditis as well as, osteoarticular, skin and soft tissue, pleuropulmonary, and device-related infections [3,39]. 

In the present study, 20 strains (10 *S. aureus* and 10 *S. epidermidis*) recovered from patients with device-associated infections, all susceptible to daptomycin and bacteriophage K, were investigated. Two *S. aureus* and seven *S. epidermidis* were methicillin resistant, whereas 14 (eight *S. aureus* and six *S. epidermidis*) produced biofilm. Both *S. aureus* and *S. epidermidis* have a pathogenic potential that stems from their ability to express a wide variety of virulence factors. Among these, our study has focused on their ability to form biofilms [1,2,40]. The presence and expression of genes, such as the *ica* operon (*icaA*, *icaD*, *icaB*, and *icaC*) and the adhesins’ encoding genes *fnbA* and *sasG* in *S. aureus* and *fbe* and *atlE* in *S. epidermidis* are associated with biofilm formation [2,4,5,19,22]. Biofilm formation was associated with *ica* carriage since 10 out of fourteen biofilm positive strains carried *ica*, whereas none of the biofilm negative isolates carried the operon in accordance with previous studies [41]. Promotion of the attachment to a surface is mediated by adhesins of the MSCRAMMs family [42]. As shown in Table 1, most isolates of our collection carried at least one adhesin gene. *FnbA* was the predominant adhesin among *S. aureus*, as published [41], whereas *S. epidermidis* carried equally *fbE* and *atlE*, especially among the biofilm positive isolates. The major cell-wall autolysin of *S. epidermidis* has been shown to enhance the release of extracellular DNA which plays a key role in the initial microbial attachment to a surface and the subsequent biofilm formation [43]. Methicillin resistance plays an important role in the pathogenic potentiality of staphylococci. Individuals with MRSA colonization or carriage have an increased risk of subsequent infection and are an important source of person-to-person transmission [7]. 

Within the attempt to restrain infections caused by these bacteria, several studies have been conducted using different antimicrobial agents, including daptomycin. A number of reports has shown that daptomycin remains very active against staphylococci, however, daptomycin non-susceptible isolates have been rarely detected worldwide, with the mechanism being attributed to various gene mutations (mprF, yycFG, RNA polymerase subunits), changes in membrane fluidity and cell wall thickness, and alterations in membrane charge [44,45]. The efficacy of daptomycin against Gram-positive bacteria, including staphylococci has also been demonstrated [46,47]. Activity against staphylococcal biofilms has been studied in a large variety of in vitro or animal models in an attempt to identify the best therapeutic options. These studies focus on the viability of bacterial cells, showing that daptomycin has satisfying results on both MSSA and MRSA strains [47,48,49,50,51,52]. In the current study, daptomycin’s efficacy was tested under biofilm forming conditions by the CV method, whereas by the MTT, only the presence of viable cells was determined. By this latter methodology, the reduction of tetrazolium salts from colorless or weakly colored aqueous solutions to brightly colored formazan is the basis of their use as vital dyes in biochemical applications [53]. Using four concentrations of daptomycin, higher activity was shown with medium and high concentrations of the drug. No difference of the antimicrobial effect of daptomycin was found regarding the bacterial species, as both *S. aureus* and *S. epidermidis* were equally susceptible to the various drug concentrations tested. It was interesting that according to our results, strains with MICs 0.75–1 mg/L were more vulnerable to the action of DHC (2 mg/L) and DMC (1 mg/L) than the strains showing lower MIC to the drug, under biofilm-forming conditions, suggesting that daptomycin is active in embedded cells. However, more strains must be tested to verify this discrepancy. 

There have been several studies in the past that demonstrate the potential use of bacteriophages against bacterial infections and their ability to disrupt biofilms, due to an increasing resistance rate to antibiotics caused by an overuse worldwide [54,55,56,57,58]. The activity of bacteriophage K against staphylococci has been also documented by Lungren et al. who demonstrated a significant decrease in *S. aureus* biofilm formation on the surface of central venous catheter material treated with bacteriophage, compared with untreated controls in an in-vitro model of central venous catheters [59]. In our study, all strains were susceptible to bacteriophage K and were classified as medium susceptible (80–100 pfu) or highly susceptible (>100 pfu). The effectiveness of phage was significantly higher in the highly susceptible strains, as expected, by both methodologies used (CV and MTT).

The combination of bacteriophage and daptomycin is a promising strategy against bacterial infections. According to Morrisette et al., phage to strain specificity contributed to synergy with antibiotics by time-kill analyses and was associated with lower development of phage resistance in *Enterococcus faecium* strains with varying susceptibilities to daptomycin and phage [60]. Our results also showed that the combined use of phage and daptomycin resulted also in increased activity against staphylococci. Specifically, the combination of low concentrations of phage and daptomycin showed similar results with either daptomycin or phage used in high concentrations, whereas, the combination of both agents in high concentrations demonstrated the best results. 

Concerning the growth curve assay results, there is not much data in the literature. Most traditional models are able to describe classical microbial growth curves. However, in the real world, a habitat that is depleted of its material resources or contains lethal agents, such as antimicrobials, cannot support a large population indefinitely and at some point, mortality takes over and reduces the population. In fact, under such conditions, mortality may prevail even before a stationary phase is reached, resulting in a clearly distinct growth peak. A common approach in the literature is the assessment of microbial growth and microbial mortality as separate phenomena and their description with different kinetic models. In this study, we followed the concept of growth–mortality duality, proposed by Horowitz et al., for modeling microbial populations, based on both cell division and cell mortality [35]. We chose a six-parameter model which can fit our data to assess the effect of the antimicrobial agent on our staphylococci population. The results showed that the isolates were classified in three groups depending on the bacteriophage effect, using the time point where the probability of mortality becomes higher than that of the bacterial proliferation; out of the 20 strains, nine were affected by both phage concentrations used, whereas three were affected only by the high concentration. Highly susceptible strains were at higher odds to be inhibited by the phage as compared to medium susceptible isolates (O.R. = 10.89, 95% C.I. 1.75–60.50). These results suggest that the application of phages as antimicrobials must be based on previous susceptibility testing. Bacteriophage’s effectiveness was achieved in approximately 3.8 h using the low concentration and in 2.4 h with the high concentration. Duarte et al., investigated the combination of a virulent bacteriophage and a phage-derived lytic protein against *S. aureus* biofilms, showing promising results of the combination according to time–kill curves and confocal microscopy results [61]. By the growth curve assay, it was not possible to study the activity of daptomycin, since no differences were observed in the ODs. We assume that this result is due to the fact that daptomycin, by its mode of action, does not cause complete cell lysis as a bacteriophage does, but only cell’s membrane damage, and therefore no significant differences were detected. 

The present study has some limitations; the first is that we have not applied methodologies—such as transmission electron microscopy, fluorescent microscopy, or colony forming units counting. However, all three applied methodologies are widely used to study biofilms and survival of bacteria with accurate results. Secondly, in our experiments we have used a limited gradient of anti-staphylococcal agents’ concentrations and therefore, no exact data about synergistic or additive effect against the tested strains could be obtained.

## 5. Conclusions

In vitro anti-biofilm activity of daptomycin was demonstrated to be higher when the effect of medium and high concentrations of the drug were tested by CV and MTT assays, whereas bacteriophage K activity was higher for the strains expressing higher susceptibility to the phage. The combination of both antistaphylococcal agents in high concentrations demonstrated the best results. By the growth curve assay, we have shown that bacteriophage’s effectiveness was achieved early (in 3.8 h or 2.4 h) depending on the susceptibility level of the strains. Even though no statistically significant difference was detected, a tendency of biofilm-negative strains to be more vulnerable to phage was also observed. Further studies are needed in order to evaluate whether combination of phages with antibiotics might be a successful choice for an alternative biofilm-associated infections’ therapy, based on the susceptibility of strains.

## Figures and Tables

**Table 1:** Phenotypic and genotypic characteristics of *S. aureus* and *S. epidermidis* studied strains. MR: methicillin-resistant; MDR: multi-drug resistant; MIC: Minimum inhibitory concentration; N: number of strains.

Species	Biofilm Formation	MR	MDR	*ica*-Positive	*fnbA*-Positive	*sasG*-Positive	*fbe*-Positive	*atlE*-POSITIVE	Bacteriophage Susceptibility		Daptomycin Susceptibility	
									Medium (80–100 pfu)	High (>100 pfu)	MIC < 0.75 mg/L	MIC 0.75–1 mg/L
		*N*	*N*	*N*	*N*	*N*	*N*	*N*	*N*	*N*	*N*	*N*
*S. aureus* (*N* = 10)	Positive (*N* = 8)	1	1	4	8	6	-	-	3	5	5	3
	Negative (*N* = 2)	1	0	0	1	1	-	-	1	1	2	0
*S. epidermidis* (*N* = 10)	Positive (*N* = 6)	4	4	6	-	-	6	6	4	2	3	3
	Negative (*N* = 4)	3	3	0	-	-	1	1	2	2	1	3
*Total*	*20*	*9*	*8*	*10*	*9*	*7*	*7*	*7*	*10*	*10*	*11*	*9*

**Table 2:** Associations of growth inhibition caused by bacteriophage with features of staphylococci.

Variable		O.R. ^1^	95 % CI ^2^	*p*-Values
Species	*S. epidermidis*	1.0		
	*S. aureus*	4.44	0.69–28.59	0.117
Bacteriophage concentration	Low concentration (PLC)	1.0		
	High concentration (PHC)	2.74	0.52–14.48	0.236
Biofilm formation	Positive	1.0		
	Negative	7.52	0.71–79.26	0.093
Susceptibility to phage	80–100 pfu	1.0		
	>100 pfu	10.29	1.75–60.50	0.010
Daptomycin MIC	<0.75 mg/L	1.0		
	0.75–1 mg/L	0.81	0.11–6.03	0.840

^1^ OR: Odds Ratio; ^2^ CI: Confidence Interval.

## Supplementary Materials

The following are available online at https://www.mdpi.com/article/10.3390/microorganisms9091853/s1, Figure S1: Effectiveness of interventions on ODs in ascending order, by CV and MTT assays. Figure S2: Representative growth curves of strains.
