# Supplementary material for: In Vitro Anti-Biofilm Activity of Bacteriophage K (ATCC 19685-B1) and Daptomycin against Staphylococci
Source: Microorganisms. 2021 Aug 31;9(9):1853. doi: 10.3390/microorganisms9091853 (PMC8468654; doi:10.3390/microorganisms9091853)
Supplement: Supplementary file 1 [file microorganisms-09-01853-s001.zip › microorganisms-1343568-supplementary.pdf]

Supplementary material

a

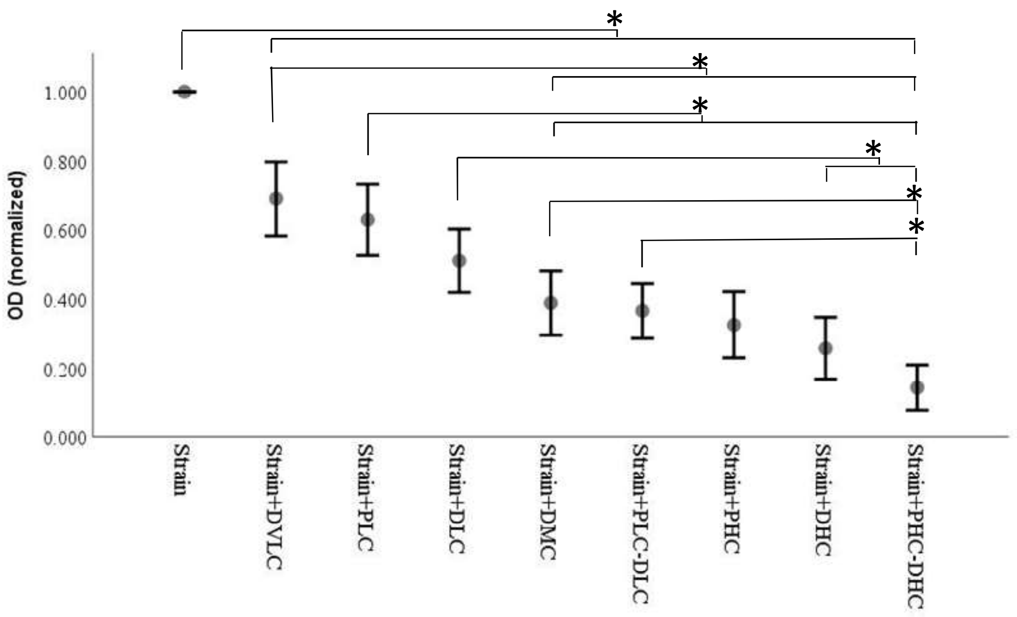

b

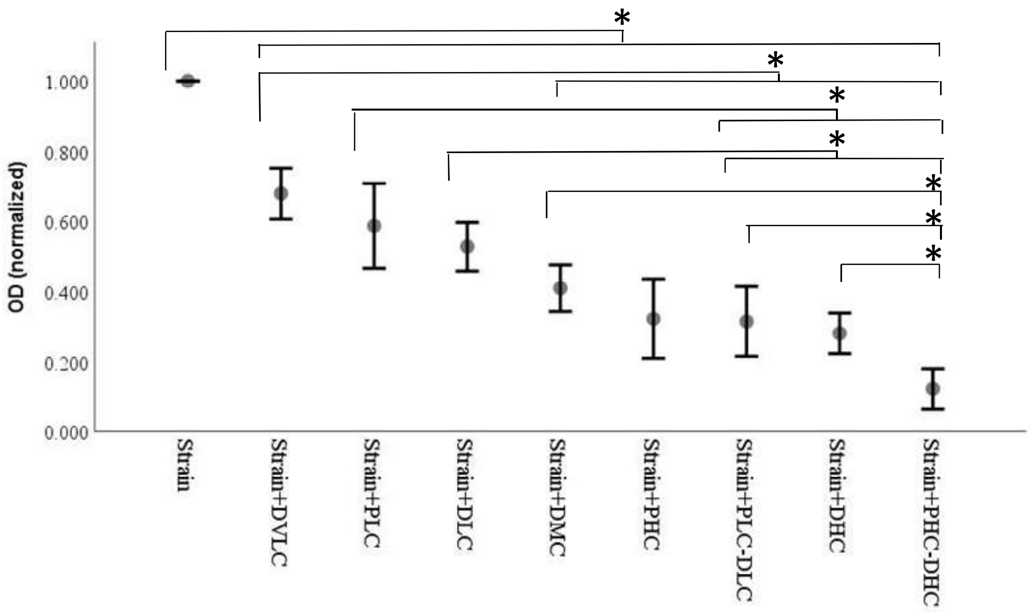

Figure S1. Effectiveness of interventions on ODs of all strains in ascending order by (**a**) the CV assay and (**b**) the MTT assay. Differences of staphylococci growth reduction were assessed by two-way ANOVA, followed by Games-Howell post-hoc tests. Error bars indicate mean OD ± 2 standard error. The brackets show significant differences between the group indicated by the start of the bracket and the group (and the following groups) indicated by the end of the bracket. PLC: 10^4 pfu/mL (phage low concentration); PHC: 10^6 pfu/mL (phage high concentration); DVLC: 0.1 mg/L (daptomycin very low concentration); DLC: 0.5 mg/L

(daptomycin low concentration); DMC: 1 mg/L (daptomycin medium concentration); DHC: 2 mg/L (daptomycin high concentration).

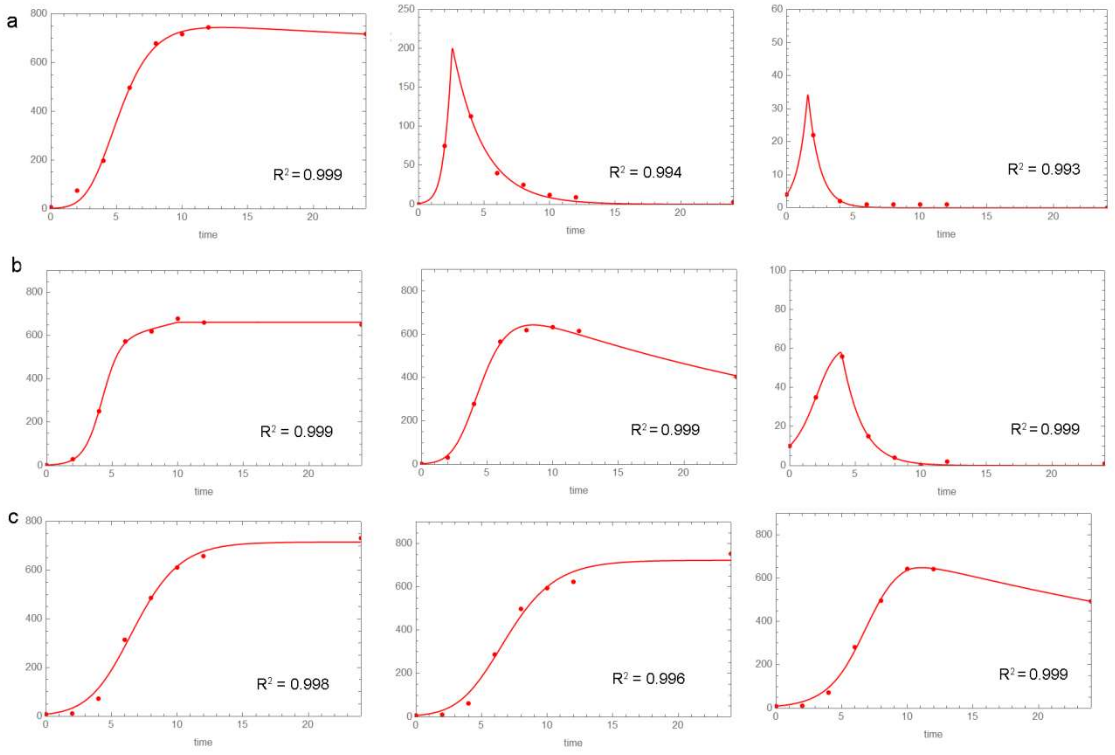

Figure S2. Representative growth curves of three strains: strain in TSB(left), strain + PLC (10^4 pfu/mL, center), strain + PHC (10^6 pfu/mL, right); (**a**) *S. aureus*, biofilm-positive,both concentrations of bacteriophage produced inhibitory effects, (**b**) *S. aureus*, biofilm-positive, only the high concentration of bacteriophage produced inhibitory effects, (**c**) *S. epidermidis*, biofilm-negative, no inhibitory effect was induced by bacteriophage.
